# Genetic Variants and Drug Efficacy in Tuberculosis: A Step toward Personalized Therapy

**DOI:** 10.1055/s-0042-1743567

**Published:** 2022-02-25

**Authors:** Almas Khan, Mohammad Abbas, Sushma Verma, Shrikant Verma, Aliya Abbas Rizvi, Fareya Haider, Syed Tasleem Raza, Farzana Mahdi

**Affiliations:** 1 Department of Personalized and Molecular Medicine, Era University, Lucknow, Uttar Pradesh, India; 2 Department of Biochemistry, Era's Lucknow Medical College and Hospital, Lucknow, Uttar Pradesh, India; 3 Department of Microbiology, Era's Lucknow Medical College and Hospital, Lucknow, Uttar Pradesh, India

**Keywords:** mycobacterium tuberculosis, germline variants, anti-TB drug-related toxicity, pharmacogenomics, personalized medicine

## Abstract

Tuberculosis (TB) continues to be a major infectious disease affecting individuals worldwide. Current TB treatment strategy recommends the standard short-course chemotherapy regimen containing first-line drug, i.e., isoniazid, rifampicin, pyrazinamide, and ethambutol to treat patients suffering from drug-susceptible TB. Although *Mycobacterium tuberculosis* , the causing agent, is susceptible to drugs, some patients do not respond to the treatment or treatment may result in serious adverse reactions. Many studies revealed that anti-TB drug-related toxicity is associated with genetic variations, and these variations may also influence attaining maximum drug concentration. Thus, inter-individual diversities play a characteristic role by influencing the genes involved in drug metabolism pathways. The development of pharmacogenomics could bring a revolution in the field of treatment, and the understanding of germline variants may give rise to optimized targeted treatments and refine the response to standard therapy. In this review, we briefly introduced the field of pharmacogenomics with the evolution in genetics and discussed the pharmacogenetic impact of genetic variations on genes involved in the activities, such as anti-TB drug transportation, metabolism, and gene regulation.

## Introduction

Tuberculosis (TB) continues to be a highly contagious public health threat, caused by bacillus *Mycobacterium tuberculosis* (MTB), 1 and has been ranked above human immunodeficiency virus (HIV)/acquired immunodeficiency syndrome since 2007. 2 About 10 million cases were recorded in the TB account, and 1.2 million who died were among HIV-infected people including 208,000 deaths of HIV-positive people. 2 India is leading followed by Indonesia and China, among the eight countries, contributing to two-thirds of the total global TB burden. 2 The disease remains a matter of grave concern as the graph of TB infection continues to incline in spite of highly efficacious treatment available since decades. Although with rapid diagnostic methods and treatment with the combination of drug regimens for over 50 years, the disease has evolved to high mortality and treatment failure. Drug response varies from individual to individual suffering from the same disease and on the same treatment plan, and some may experience adverse drug reactions (ADR). 3 Non-genetic factors such as age, gender, nutritional status, general medical condition (e.g., hepatic and renal physiology), lifestyle (diet, alcohol abuse, and smoking), concomitant therapy, or the presence of co-morbidity have been previously attributed to differences in the risk–benefit ratio among patients taking the same drug. Aside from these considerations, changes in patient genetic make-up are now understood to have a significant impact on treatment response. 4 These variations among individuals are due to several factors including different allele frequency distributions of single nucleotide polymorphisms (SNPs) that have a functional impact on genes association with drug response. 5 Hence, absorption, distribution, metabolism and excretion, pharmacokinetics (PK), and pharmacodynamics of drugs are influenced by a genetic variation which affects drug efficacy and drug-induced toxicity and, thus, leads to ADRs or therapeutic failure. All these factors due to variations are validated by genotypes, and therefore, pharmacogenetic implementation in the clinical setting has become an important aspect of targeted therapy. 6 Pharmacogenomics research has a long-term purpose of the development of individualized medication based on the patient's genetic sequence to achieve maximum response and avoid undesirable drug responses. 7

Isoniazid (INH), rifampicin (RF), pyrazinamide, and ethambutol are the first-line anti-TB drugs currently recommended by World Health Organization. 2 INH and RF are the two key drugs used for the treatment of TB. Resistance offered to these drugs by the mycobacteria or adverse reaction caused by the drugs results in treatment prolongation from 6 to 9 months 8 or sometimes treatment has to be stopped due to the excessive damaged caused by the adverse reaction. 9 Efficacy and early antibacterial activity of the drugs are related to the dose or PK; therefore, the variation in the PK of INH and RF influences the clinical consequence of TB treatment. 9 With beneficial effects, there are various adverse effects especially peripheral neuropathy and hepatotoxicity induced by INH therapies. 10 11 We have outlined the emerging role of pharmacogenomics in this review and the way in which valuable tools for the determination of inter-individual variation are found. In this review, we focus on genes involved in RF and INH transport and metabolism, as well as the genes governing the transcription of transporter and metabolizing genes.

## Genetics and Individualized Treatment (Personalized Medicine)

**Fig. 1:**
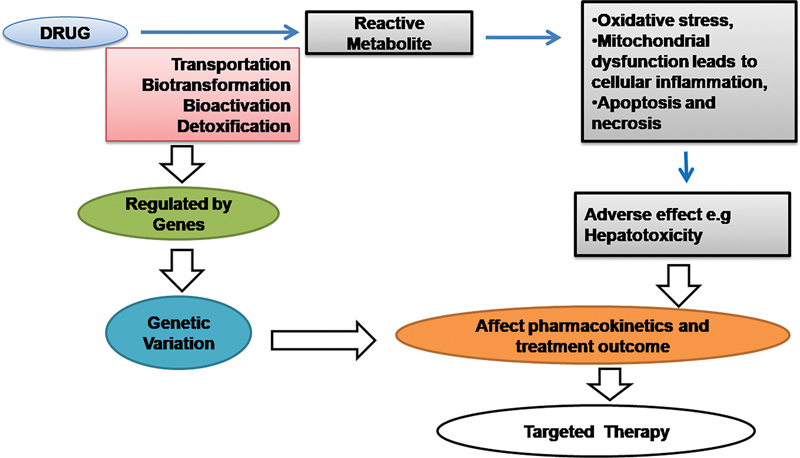
Concept of genotyping-based targeted/individualized therapy.

The idea of individualized medicine is an appealing concept for the future of treatment. It is divine to employ molecular research data to categorize disease and its susceptibility, aid the development and rationale of new therapeutic regimen, 12 and help in patient treatment with greater specificity and potency with fewer side effects 13 ( Fig. 1 ).

With the advancement in technology and development in biomedical sciences, research findings have unfolded several predictive sciences. Pharmacogenetics and pharmacogenomics are two among new predictive sciences that have emerged from the knowledge discovered in biomedical research. The research in these fields could promise a crucial step toward personalized treatment. 14 It is necessary to understand the disposition and fate of drugs, i.e., PK and pharmacodynamics of drugs which include therapeutic outcomes with adverse effects. The PK and pharmacodynamics of drugs are determined by complicated processes involving the majority of proteins coded by a variety of genes, which determine drug transport, metabolism, potency, and drug detoxification. Variations in the coding region may induce amino acid substitution at the specific location of protein which consequently alters protein function. If the variation is found in the regulatory region, it may affect transcription and translational mechanisms with gene product modulation (mRNA and proteins) and their expression levels may differ. 4 15 Variations in DNA sequence in population at or above 1% allelic frequency are termed as polymorphism, whereas variations with lower frequencies are defined as mutations. Metabolic activities or drug affinity to its receptor and efficacy depends on enzymes coded by mutated or polymorphic genes, which can alter pharmacological response in individuals or in some ethnic groups, within population. Single-nucleotide polymorphisms (SNPs) are a type of genetic variants that occur mostly as a result of the substitution of a single base pair and they are commonly known. 4 15 Studies revealed that variability among individuals in PK vulnerability to drug accounts for some unfortunate outcomes, also in those patients who do not miss doses. This has challenged the conventional concept that therapeutic failure, reoccurrence, and the emergence of antimicrobial resistance are primarily due to poor adherence. 16

The severe and ubiquitous challenge in the TB management of patients is PK heterogeneity. *SLCO1B 1, ABCB1, PXR, CAR* , and *CES* genetic variations have drawn scientific attention because they have an impact on a broad spectrum of medications' PK. 17 Genetic changes in the acetylation status are linked to marked inter-individual variation in circulating INH concentration and clearance after medication. The differences in INH inactivation and elimination rates in various (fast and slow) acetylation phenotypes are principally attributable to differences in the rate of INH acetylation in the liver and small intestine by a genetically regulated polymorphic N-acetyltransferase (NAT) enzyme. 18

## Rifampicin

**Fig. 2:**
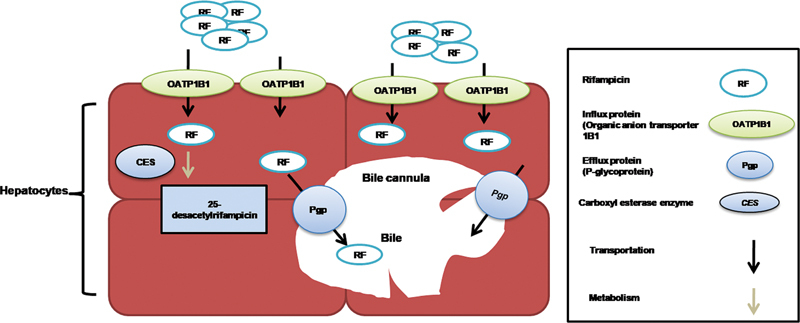
Diagram showing the uptake, transformation, and efflux of rifampicin in hepatocytes.

RF proved to be a cornerstone TB treatment by shortening the period of anti-tubercular therapy (ATT) from 18 to 9 months and enhanced recovery rates when it was introduced in combinational chemotherapy for TB during 1960s. 19 20 RF diffuses freely in the tissues, living cells, and bacteria which make it easily available against the intracellular pathogens like MTB. It exhibits its anti-mycobacterial activity by arresting the RNA synthesis from MTB's DNA through the β-subunit of RNA polymerase (RNA pol). 21 RF action drives through the rpo β gene which codes for the β-subunit of RNA pol, and mutation in the rpo β gene is the reason behind the development of more than 95% of resistance against RF. Major mutation hotspots are found in 81bp-RF resistance-determining region (RRDR). Commonly known mutated codons in RRDR are rpo β 531, rpo β 526, and rpo β 516. 22 Its antibacterial properties and resistance development are influenced by drug bioavailability (concentration), and higher doses of 1,200 mg or more daily may be effective. 23 As a result, increasing the dose of RF from the standard dosing based on weight may aid in achieving desired plasma PK and pharmacodynamics. 24 Few recent studies have shown that the high dosage of RF could result in better therapy outcomes in patients. 25 26 Many studies reported a correlation between various genetic variants and significant changes in RF plasma levels in TB patients. 27 RF hepatocellular uptake is typically performed by organic anion-transporting polypeptide 1B1 (OATP1B1) 28 and metabolism is catalyzed by hepatic β esterases and arylacetamide deacetylase 29 to its active metabolic form, 25-desacetylrifampicin, and then, it is excreted via bile and renal routes after first pass metabolism. 30 OATP1B1 is main among the major influx transporter proteins, a 691 amino acid protein coded by solute carrier (SLC) organic anion transporter family member 1B1 ( *SLCO1B1* ) 31 gene that predominantly presents at the basolateral membrane of hepatocytes 32 and modulates the hepatic uptake of drug from bloodstream. 33 OATP1B1 transporter protein has a strong affinity for RF. 34 Membrane drug transporter superfamilies include SLC and adenosine triphosphate (ATP)-binding cassette (ABC) transporters. 32 Around 400 transporters in membrane belong to SLC and ABC superfamily, with approximately 32 of them being clinically linked and potentially important drivers of drug PK and individual drug responses. 35 The sinusoidal inflow transporter SLCO1B1 and the efflux transporter ABCB1 influence RF distribution 36 while absorption in liver and biliary excretion. 37 Fifteen exons and 190 known polymorphisms having minor allele of more than 5% frequency are found in the *SLCO1B1* gene. 38 rs4149056 625T > C and rs2306283 492A > G are two well-characterized variation in the *SLCO1B1* gene 39 ( Fig. 2 ).

According to studies, a common single nucleotide variant (rs4149056 c.521T > C p. V174A) reduces SLCO1B1 expression, resulting in lower OATP1B1 uptake/transport activity and higher plasma levels. 40 Allegra et al reported high RF plasma concentration in TB patients with the SLCO1B1 rs4149056 variant, and analysis by multivariate linear regression also suggested that the SLCO1B1 rs4149056 variant can be considered as a positive predictive marker for raised RF concentration. 41 Weiner et al observed decreased RF exposure in patients who had SLCO1B1 rs11045819 (c.463C > A p. Pro155Thr) polymorphism. 42 When compared with patients with the wild-type allele (CC), patients having rs11045819 variant allele (CA) in SLCO1B1 showed 42% low RF exposure (25.6 µg*h/mL), 34% reduced peak concentration (5 µg/L), and 63% detectable oral clearance (22 L/h). 42 The functional effects of variation rs2306283 (c.388A > G p.Asn130Asp) have been observed to be inconsistent. 40 43 A study by Dompreh et al observed that rs2306283 variant in SLCO1B1 was found to be related to decreased RF concentration in pediatric TB patients. In paired analysis, patients having SLCO1B1 homozygous variation (AA) exhibited significantly lesser RF concentration than patients having wild genotype (GG). 44 In the African population, there is an elevation in the prevalence of the SLCO1B1 rs4149032 polymorphism in intron 2 haplotype tagging SNP (tSNP) which is associated with lower RF exposure. 45 Patients with heterozygous and homozygous mutations had considerably lower RF bioavailability, with 18 and 28% declines in RF bioavailability, respectively. 46

RF is a substrate of P-glycoprotein (Pgp) efflux pump. 47 Pgp is a multi-domain integer membrane protein that uses ATP energy to move solutes/ions across the membranes in eukaryotes (efflux pump). 48 49 50 Pgp is the ABCB1 transporter protein constituting of 1,280 amino acids that is encoded by the ABC gene. 51 The ABCB1 gene is present on chromosome number 7 and has 29 exons in a 251.3-kb genomic area. The most prevalent SNPs in this ABCB1 gene are rs1128503, rs2032582, and rs1045642. 52 rs1128503 and rs1045642 variants are synonymous, while missense mutation is found in rs2032582. 53 There was no statistically significant correlation between ABCB1 variants and RF PK in any of the studies. However, only few of ABCB1 variants were studied in relation to RF exposures. Rare ABCB1 mutations that could affect medication PK have yet to be thoroughly investigated in terms of their functional consequences. 52 The transcription of SLCO1B1 and ABCB1 genes is regulated by *PXR* and *CAR* genes. *PXR* and *CAR* are nuclear receptors in the group I family that regulate a variety of transcriptions, including those of medicinal enzymes and drug transporter genes. 52 Few research studies have looked into the likelihood of SNPs in these genes being linked to RF plasma levels. The *PXR* gene has rs2472677 and rs1523130 variations in the intron 1 and 50UTR regions, respectively. These areas illustrate the transcription factor binding sites of regulatory regions of *PXR* . 53 rs2307424 variant is due to synonymous mutation in the *CAR* gene (c.540 C > T). 54 None of the variants have found to be associated with the RF plasma level and its exposure.

B-esterase is the enzyme that converts RF to 25-desacetylrifampin. 55 The carboxylesterase (CES), acetylcholinesterase, and butyrylcholinesterase enzymes are all members of the B-esterase family. CES is a broad substrate-specific enzyme that hydrolyzes ester, thioester, amide, and carbamate bonds, which is implicated in the metabolism of various endobiotic and xenobiotic substances. The two primary isoenzymes of human CES, mainly expressed in the liver, are CES1 and CES2, which are coded by the *CES1* and *CES2* genes, respectively. 56 Several genetic variants of *CES1* and *CES2* have been associated with a significant difference in drug therapy responses over the past decades. As a result, comparing the PK of substrate medicines to genetic variations in these genes becomes important. 57 Both *CES1* and *CES2* genes lie on chromosome 16 and have 14 and 12 exons, respectively. 58 Sloan et al found that rs12149368 mutation in the exon 1 (5′UTR) region in *CES1* gene has no effect on plasma RF concentration. 16 Song et al observed 10 SNPs: the promoter region variants c-2548C > T and c-2263A > G, as well as c.269–965A > G, c.474–152T > C, c.615 + 120G > A, c.1612 + 136G > A, and c.1613–87 G > A variants in intronic region and c.1872*69A > G, c.1872*302_304delGAA, c.1872*445C > G variants in 3′UTR within the RF level. The *CES2* c.-22263A > G variation was linked to elevated concentration of RF in plasma in TB patients. Plasma RF concentrations in homozygous major, heterozygous, and homozygous minor allele were 8.9 ± 2.9, 10.5 ± 3.1, and 13.9 ± 7.4 mg/L, respectively, in homozygous major allele, heterozygous, and homozygous carrying minor allele. The shift of G from A in the *CES2* c.-22263A > G variant has been found linked with continuous fall in activity of luciferase, this may lead to low metabolism and higher plasma RF levels, according to the study. 59 Dompreh et al, on the contrary, found no differences in exposures of RF with *CES2* rs3759994 variation. 9

## Isoniazid

Due to low molecular weight and high water solubility, 18 INH can be easily absorbed from the gastrointestinal tract (GIT) 4 60 ; thus, the peak plasma concentration reaches in 1 to 3 hours of drug intake. 61 INH reaches all tissues 62 and body fluids including cerebrospinal fluid, saliva, pleural, and peritoneal fluid 63 and to lungs after absorption from GIT. 64 65 INH also attains peak concentration in the breast milk of lactating mother within 1 hour of drug administration. 66

INH metabolism mainly follows enzyme-dependent pathways such as acetylation through NAT2 enzyme and hydrolysis catalyzed by acyl amidase. 67 INH-NAD ^+^ adducts is also formed by the combined action of human neutrophil myeloperoxidase and catalase-peroxidase ( *KAT G* ) of MTB. 68 69

INH is initially metabolized by a non-inducible hepatic and intestinal enzyme known as the NAT type 2 (NAT2) 67 which is coded by highly polymorphic gene called *NAT 2* gene. 70 INH is acetylated to acetylisoniazid by NAT2 enzyme 2, and it is also hydrolyzed to form isonicotinic acid (INA) and hydrazine (Hz) through the amidase enzyme. Acetylisoniazid can also be hydrolyzed to produce INA and acetylhydrazine (AcHz); furthermore, Hz can be converted into AcHz and diacetylhydrazine via acetylation catalyzed by NAT2 enzyme. 71 Hz and AcHz are supposed to be converted into reactive metabolites through oxidation and may be responsible for the INH hepatotoxicity which can be mediated by microsomal P450s like *CYP2 E* . 67 72

Arylamine NAT (EC2.3.1.5) is the cytosolic enzyme of 30 kDa found in almost every species, both in prokaryotes and in eukaryotes. 73 It is present in multiple isoenzymes forms, 74 and its two distinct isoforms, NAT1 and NAT2, with overlapping substrate specificities have been studied in humans. 75 Biotransformations of xenobiotics are mainly catalyzed by NAT1 and NAT2. 74 NAT2 expression is confined to liver and GIT, whereas NAT1 is expressed in the majority of the tissues along with endocrine tissues, blood cells, neural tissues, as well as in liver and GIT. 76 Being a transferase group of enzyme, it inactivates the arylamine and Hz-based xenobiotics by transferring acetyl group of acetyl CoA to the terminal nitrogen atom of the xenobiotics. 18 Hence, it is accountable for Hz drug acetylation and many carcinogenic aromatic amines along with endogenous molecules such as serotonin. 67

NAT1 enzyme is limited to few specific substrates (p-aminobenzoic acid), whereas NAT2 enzymes 76 play a crucial role in the metabolism of a wide variety of drugs like dapsone, sulfadoxine, INH, procainamide, and hydralazine along with chemicals which are present in the diet. 77 *NAT1* and *NAT2* genes encode NAT1 and NAT2 enzymes, respectively. 78 The *NAT2* is autosomal dominant and intronless having a single open reading frame of 870 base pairs, located on chromosome 8p22. Variations in *NAT2* result in slow, intermediate, or fast acetylation phenotypes with broad inter-ethnic groups. NAT2 confer slow, intermediate, or fast acetylation phenotypes with broad interethnic variations. There are 53 *NAT2* alleles presently known, and each allelic variant exhibits the combination of one, two, three, or four nucleotide alteration. There are seven missense mutations (G191A, T341C, A434C, G590A, A830G, A845C, and G857A) and four silent mutations (T111C, C282T, C481T, and C759A) within the coding region. 5 The NAT2*4 is a wild-type allele, does not have any nucleotide substitution, and is known to be associated with the fast acetylation phenotype. This acetylation phenotype can be predicted by genotyping with 95% accuracy.

Considering genetic characteristics of NAT2 enzymes, the ability of drug and exogenous compound acetylation and inter-individual variation among the population is widely related. 79 As NAT2 enzyme is a dominant catalyzer in INH biotransformation (formation of AcINH), bio-activation (formation of AcHz) and detoxification (formation of DiAcHZ), due to different allelic distributions of *NAT2* gene, results in variation in the acetylation profile of drugs in the same population. 67 70 The degree of acetylation has been associated with a higher risk of INH-induced hepatotoxicity in various studies. There are three different phenotypic acetylation profiles based on SNPs in the exon of the *NAT2* gene. Individuals with slow NAT2 acetylation allele have a low acetylation rate, resulting in a higher plasma concentration of the parent drug and possibly better efficacy. However, individuals with slow acetylation may experience adverse effects due to the accumulation of toxic metabolites such as AcHz during the ongoing metabolism of INH and toxic metabaolites contributing to hepatitis threat. Fast acetylation causes low plasma drug concentrations, making them less toxic and also less effective, while intermediate acetylation leads to in-between results. 80 Alleles of slow acetylation have been found to be associated with increased risk of INH hepatotoxicity in numerous clinical investigations. 67 Individuals with slow acetylation had higher plasma levels of INH and AcHz than those with fast acetylation. According to Donald et al, in slow acetylation of INH, 3 mg/kg dose is sufficient to attain the expected therapeutic objectives of anti-TB treatment, but in the case of fast acetylation, a 6 mg/kg of dose is required to provide adequate bactericidal activity. 81

## Conclusion

The genetic and molecular research has proved to be a cornerstone in personalized medicine and is indicative of its expanding importance in the field of health care. Heterogeneous drug response with anti-tubercular drug therapy is a severe problem in TB patients. So, here we emphasized the genetic association with the PK of ATT drug. Genetic polymorphism in drug transporter genes, regulatory genes like *SLCO1B, ABCB1, PXR* , and *CAR* and drug-metabolizing genes such as *CES* and *NAT2* which drive the response, has been found to be of keen interest to rule out disease predisposition. By implementing genotyping assays prior to treatment administration, clinicians could better determine the dose which could be the main prospect of precision medicine. Additional studies are required to gain the core knowledge of drug fate association with genetic variations within the population. With advances in knowledge and findings, pharmacogenetics and pharmacogenomics will have a greater impact on drug research and development, clinical trials, and clinical practice.
